# Reverse Evolution: Driving Forces Behind the Loss of Acquired Photosynthetic Traits

**DOI:** 10.1371/journal.pone.0008465

**Published:** 2009-12-29

**Authors:** Francisco de Castro, Ursula Gaedke, Jens Boenigk

**Affiliations:** 1 Department of Ecology and Ecosystem Modelling, University of Potsdam, Potsdam, Germany; 2 Institute for Limnology, Austrian Academy of Sciences, Mondsee, Austria; Mt. Alison University, Canada

## Abstract

**Background:**

The loss of photosynthesis has occurred often in eukaryotic evolution, even more than its acquisition, which occurred at least nine times independently and which generated the evolution of the supergroups Archaeplastida, Rhizaria, Chromalveolata and Excavata. This secondary loss of autotrophic capability is essential to explain the evolution of eukaryotes and the high diversity of protists, which has been severely underestimated until recently. However, the ecological and evolutionary scenarios behind this evolutionary “step back” are still largely unknown.

**Methodology/Principal Findings:**

Using a dynamic model of heterotrophic and mixotrophic flagellates and two types of prey, large bacteria and ultramicrobacteria, we examine the influence of DOC concentration, mixotroph's photosynthetic growth rate, and external limitations of photosynthesis on the coexistence of both types of flagellates. Our key premises are: large bacteria grow faster than small ones at high DOC concentrations, and *vice versa*; and heterotrophic flagellates are more efficient than the mixotrophs grazing small bacteria (both empirically supported). We show that differential efficiency in bacteria grazing, which strongly depends on cell size, is a key factor to explain the loss of photosynthesis in mixotrophs (which combine photosynthesis and bacterivory) leading to purely heterotrophic lineages. Further, we show in what conditions an heterotroph mutant can coexist, or even out-compete, its mixotrophic ancestor, suggesting that bacterivory and cell size reduction may have been major triggers for the diversification of eukaryotes.

**Conclusions/Significance:**

Our results suggest that, provided the mixotroph's photosynthetic advantage is not too large, the (small) heterotroph will also dominate in nutrient-poor environments and will readily invade a community of mixotrophs and bacteria, due to its higher efficiency exploiting the ultramicrobacteria. As carbon-limited conditions were presumably widespread throughout Earth history, such a scenario may explain the numerous transitions from phototrophy to mixotrophy and further to heterotrophy within virtually all major algal lineages. We challenge prevailing concepts that affiliated the evolution of phagotrophy with eutrophic or strongly light-limited environments only.

## Introduction

Why would a photosynthetic organism lose photosynthesis? The advantages of autotrophy are obvious, especially in environments with limited carbon sources. However, the secondary loss of functional chloroplasts and hence of photosynthetic ability (*i.e.* the transition from ‘plant’ to ‘animal’) has happened frequently in the evolution of eukaryotes [1], [2] and is documented for all phototrophic supergroups, with numerous examples for the whole domain of eukaryotes: the Archaeplastida [3], the Excavata [4] and the Chromalveolata [5]. Within the Chrysophyceae alone, the loss of photosynthesis occurred at least five times [5]. Many colorless algae lineages still possess remains of the plastid as evidence for their former life style [2], and even the malaria parasite (*Plasmodium* spp.) bears remains of a plastid [6]. This fact may be one of the main causes of the previously unrecognized diversity of protists.

The numerous colourless taxa (*i.e.* obligate heterotrophic) within many algal lineages raise the question of the evolutionary scenarios resulting in the loss of the pigmentation. Mixotrophy (*i.e.* the combination of autotrophic and heterotrophic ability in the same organism) can be advantageous over autotrophy in environments with low inorganic nutrients that limit photosynthetic growth (*e.g*. P, Fe, N), because the uptake of particulate food, such as bacteria, opens the option for alternative nutrient sources [7]. On the other hand, mixotrophy offers advantages over heterotrophy because it gives access to an additional carbon source, unless photosynthesis is strongly limited by available light. Hence, mixotrophs are considered to be superior to specialised heterotrophs and phototrophs at limiting conditions (e.g. phototrophs limited by inorganic nutrients and heterotrophs by low bacterial abundance, [8], [9]), where they can benefit from both types of nutrition. However, the metabolic costs of having both functional chloroplasts and the structures required for phagotrophy are thought to be high [10], and these high costs may account for a strong evolutionary pressure towards efficient resource acquisition. Thus, even though resource limitation may explain the evolution from autotrophy to mixotrophy, it is much less clear what would lead mixotrophs to a complete loss of chloroplasts in environments with no light limitation. Whereas the advantage of specialised heterotrophy in light-limited environments, such as soils and deep or turbid water, is intuitively clear, given the costs of mixotrophy, the potential advantages of losing photosynthesis in resource-limited but well-lit situations are not.

Previous models predicted the possibility of a bifurcation of a mixotroph into obligate heterotrophic and autotrophic lineages under some conditions [11]. However, this type of bifurcation, which includes the disappearance of the mixotrophic ancestor, would be a very unlikely evolutionary scenario.

**Figure 1:**
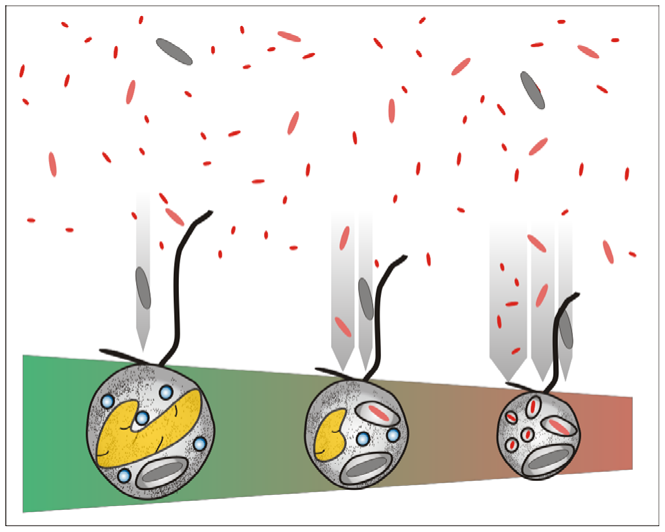
Conceptual scheme of the model. The reduction of intracellular structures is a mean to reduce the cell size of the predatory flagellates. A consequence of this size reduction is an optimized predator-prey-size ratio for the smallest flagellate when preying upon the smallest bacteria, *i.e.* ultramicrobacteria, which largely escape predation by larger flagellates (Pernthaler 19). This optimization is thus an evolutionary driving force behind the differentiation of mixotrophic algae into obligate heterotrophic flagellates.

Here we analysed the efficiency to exploit alternative carbon sources, namely differently sized bacterioplankton, as a key factor for the evolution of heterotrophic lineages from mixotrophs. The dominance of ultramicobacteria in a wide range of aquatic systems and their different grazing susceptibility to small and large flagellates suggest that size-based predator-prey interactions may play a key role in the evolution of heterotrophic algal lineages (Fig. 1). For interception-feeding flagellates of a given size, the successful prey capture and feeding rates are more strongly correlated with prey volume (∼r^3^) than diameter (∼r) , especially when differentiating between interception and filter feeders [12], [13]. Thus, even small reductions in cell size can render a large improvement in feeding efficiency on ultramicrobacteria. In general, optimal predator-prey volume ratio for interception-feeding nanoflagellates is around 1∶10 [14] and a ratio beyond 1∶500 results in inefficient feeding [13], [15]. Based on this constraint, two evolutionary pathways can, in theory, increase the flagellate's feeding efficiency on small bacteria: (i) switching from interception feeding to filter feeding and (ii) reducing the cell size to optimize the predator-prey size ratio for interception feeding. The former alternative is, in fact, realized in choanoflagellates, but seems evolutionary complicated for other flagellate lineages as it would require a complex additional set of genes to be taken up by horizontal gene transfer. The later alternative seems therefore more plausible. However, even though the smallest eukaryotes are only 1 µm in size (*i.e.* the green algae *Ostreococcus* sp. [16]), the reduction of cell size for most picoeukaryotes, and specifically mixotrophic eukaryotes, is complicated for two reasons: (i) keeping the machinery for both phototrophy and phagotrophy seems to require a larger cell size, although bacterivory has been occasionally reported for microalgae around 2 µm (*Micromonas pulsilla* [17]); and (ii) the presence of a secondary plastid requires a large additional set of genes [1] and therefore probably does not allow a dramatic reduction in genome size (except for the Archaeplastida which harbor a primary plastid). Even the exclusively heterotrophic lineages are hardly smaller than 2–3 µm [18], close to the maximum size for optimal feeding on ultramicrobacteria [15], [19]. To our knowledge, any mixotrophic chromist capable of efficient photosynthetic and phagotrophic nutrition is considerably larger than 2–3 µm.

## Methods

Based on recent evidence, we argue that the efficient grazing on small bacteria (as additional carbon source) is likely to play a role in the evolutionary step from mixotrophy to heterotrophy, and could outweigh the advantage provided by photosynthesis. Specifically, recent key observations demonstrate that:

1. Flagellates, in general, are efficient consumers of bacteria, and show strong food size selection [19]. “Large” bacteria (>0.1 µm^3^) are more susceptible to flagellate predation than ultramicrobacteria, i.e. cells <0.01 µm^3^ , which may largely escape grazing by larger flagellates [20] despite their usually high abundances.
2. Flagellates, which became heterotrophic by reducing their intracellular structures (specifically the chloroplast) can become smaller than their mixotrophic relatives that kept these structures, and so are more efficient consumers of small bacteria [21], [15]. Although within a given taxonomic group, smaller flagellates are more efficient in capturing small bacteria than larger flagellates [15], [22], it does not imply that all heterotrophic flagellates are smaller than all mixotrophic ones or more efficient bacterivores than all mixotrophic ones.
3. Large bacteria have higher growth rates than small ones at high dissolved organic carbon (DOC) concentrations [23] but small bacteria can better exploit low DOC concentrations. Consequently small bacteria usually dominate in systems with low concentrations of DOC, although they may also dominate in high DOC environments due to a weaker top-down control, as compared to larger bacteria [20], [23]. It has been shown that bacteria with high maximal growth rates tend to be poor competitors under substrate limitation and *vice versa* [24]. These growth characteristics appear to be linked to bacterial size: at low substrate concentrations small bacteria may have a certain advantage due to a larger surface/volume ratio [24], [25] whereas large bacteria can achieve higher growth rates [26]. Maximal growth rates of bacteria have been related to the copy number of ribosomal operons in the genome, *i.e*. that multiple operons, as found in the genomes of many large bacteria, are required to achieve high growth rates [27], [28]. Ultramicobacteria do often have small genomes and for a number of taxa it has been shown that they possess only a single copy of the ribosomal operon [26], [27]. This is consistent with the comparatively low maximal growth rates achieved by these bacteria [26], [29] in contrast to high growth rates achieved by large bacteria [26].

We implement this evidence into a dynamic model of competing heterotrophic and mixotrophic flagellates and two types of bacterial prey. With this model, we examined the influence of (i) carbon concentration (as a measure of trophic conditions), (ii) the photosynthetic capacity of the mixotroph, and (iii) limitation of light availability, on the probability of invasion of a heterotrophic mutant into a community of its mixotrophic ancestor and the two types of bacteria.

The model considers, beside DOC concentrations (C), four functional groups: mixotrophic flagellates (M) and a heterotrophic flagellate mutant (H), large and small bacteria (L and S, respectively):
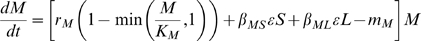
(1)

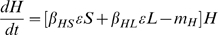
(2)

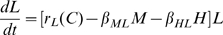
(3)

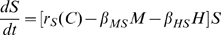
(4)

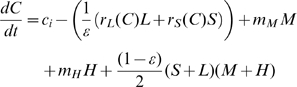
(5)

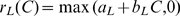
(6)

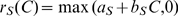
(7)

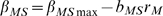
(8)

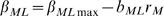
(9)where:

M: Mixotrophic flagellates in units of carbon

H: Heterotrophic flagellates in units of carbon

L: Large bacteria in units of carbon

S: Small bacteria in units of carbon

C: Dissolved organic carbon (DOC) concentration in the medium

For the definitions and values of the parameters, see Table 1.

**Table 1:** Values and description of the model parameters.

Parameter	Description	Value	Units
C_M_	Mixotrophic flagellates in units of carbon	15000	fgC/cell
C_H_	Heterotrophic flagellates in units of carbon	3000	fgC/cell
C_L_	Large Bacteria in units of carbon	77	fgC/cell
C_S_	Small Bacteria in units of carbon	19	fgC/cell
*m_M_*	Mortality rate of Mixotroph	0.008	h^−1^
*m_H_*	Mortality rate of Heterotroph	0.02	h^−1^
*m_S_*	Mortality rate of Small bacteria	0	h^−1^
*m_L_*	Mortality rate of Large bacteria	0	h^−1^
*r_L_*	Intrinsic growth rate of large bacteria. Function of carbon (>0)	r_L_ = a_L_+b_L_*C	h^−1^
*r_S_*	Intrinsic growth rate of small bacteria. Function of carbon (>0)	r_S_ = a_S_+b_S_*C	h^−1^
*r_M_*	Intrinsic photosynthetic growth rate of Mixotroph	variable	h^−1^
*K_M_*	Carrying capacity of mixotroph photosynthetic growth	variable	fgC
*a_L_*	Intercept of growth rate function of L	−0.001	ngC/nl/h
*a_S_*	Intercept of growth rate function of S	−0.001	ngC/nl/h
*b_L_*	Slope of growth rate function of L	0.0002	unitless
*b_S_*	Slope of growth rate function of S	0.0001	unitless
*c_i_*	External DOC input	variable	fgC/nl/h
*e*	assimilation efficiency	0.3	unitless
*a_2M_*	Filtration rate per unit of mass of mixotrophs (fr_M_/C_M_ )	30/15000	nl/fgC/h
*a_2H_*	Filtration rate per unit of mass of mixotrophs (fr_H_/C_H_ )	10/3000	nl/fgC/h
*a_3MS_*	Capture efficiency of Small bacteria by mixotrophs	0.15	unitless
*a_3ML_*	Capture efficiency of Large bacteria by mixotrophs	0.90	unitless
*a_3HS_*	Capture efficiency of Small bacteria by heterotrophs	0.80	unitless
*a_3HL_*	Capture efficiency of Large bacteria by heterotrophs	0.90	unitless
*b*	Rate of capture of bacteria (L or S) by flagellates (H or M)	*a*2 * *a*3	unitless
fr_M_	Max. rate of filtration of Mixotrophs	30	nl/h
fr_H_	Max. rate of filtration of Heterotrophs	10	nl/h

The subscripts M, H, L and S, for *b* have been omitted for brevity. All rates are per hour. The parameter values have been extracted from the literature and partial sensitivity analyses have been performed in order to demonstrate that the model results are not unduly sensitive to the parameter values chosen: carbon content is based on cell size measurements and conversion factors proposed by [36]. Filtration rates, capture efficiencies and assimilation efficiencies are based on: [37]–[42]. The slopes of the bacterial growth rate functions are based on [43]–[45].

The term 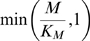 (eq. 1) is used to avoid negative growth values due to the heterotrophic nutrition by the mixotroph by which *M* could be larger than K*_M_*.

Equations 8–9 represent a trade-off in the mixotroph between photosynthetic and heterotrophic capacities, assuming a linear decrease of capture efficiency for both types of bacteria, as its photosynthetic ability increases [30]. Without this trade-off the mixotroph would become an unrealistic “super star”, performing best in any conditions, which renders it impossible for the heterotroph to compete, unless photosynthesis is severely limited by external factors (*i.e.* very low K*_M_*). Importantly, our initial assumption that mixotrophs are much less efficient than heterotrophs grazing small bacteria due to their larger size, can be relaxed to a considerable extent and still the conclusions from the model hold (Text S1, Fig. S1).

We performed a partial sensitivity test of the model, including three parameters: mixotroph's photosynthetic growth rate (*r_M_*), mixotroph's photosynthetic carrying capacity (K*_M_*), and external carbon input (*c_i_*). The first two parameters define the photosynthetic advantage of the mixotroph, while the third controls the levels of DOC in the medium, reflecting the trophic state of the habitat. For the sensitivity test, the values for *r_M_* ranged from 0.001 to 0.016 (h^−1^). The values of K*_M_* and *c_i_* ranged from 0.1 to 5 (fgC/nl). For each combination of values the model was run with a community comprised of mixotrophs and both types of bacteria for 1000 hours (app. 1.5 months), to allow the community to develop. Then, the heterotrophic mutant was introduced at low density (a hundredth of the final mixotroph's density) and the model was run until a stable situation was reached (*i.e.* the coefficient of variation for all the state variables over the last 1000 hours was lower than 10^−4^).

## Results

**Figure 2:**
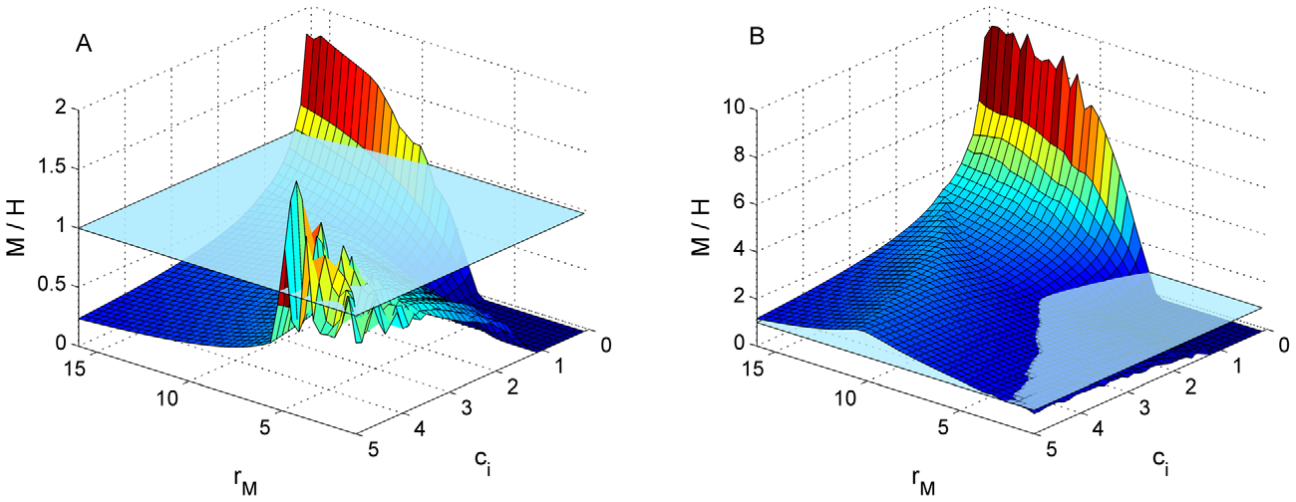
Relative dominance of Mixotrophs vs. Heterotrophs. Ratio of Mixotroph biomass (M) to Heterotroph biomass (H) at the equilibrium, as a function of carbon input (*c_i_*) and photosynthetic growth rate (*r_M_*×1000). Left panel (A), represents low light availability (*K_M_* = 0.5), while right panel (B) represents high light availability (*K_M_* = 2.5). The horizontal plane marks the 1∶1 ratio. Note the different scale in both panels. At a relatively high carrying capacity (B), the mixotrophs dominate the community for most of the range of carbon input (*c_i_*) and growth rate (*r_M_*). Only in a very carbon-poor environment and with a low photosynthetic ability can the heterotroph reach higher biomass. The mixotroph dominance is, again, particularly strong at the combination of low *c_i_* and high photosynthesis. However, as in the previous case, at high photosynthetic rates the dominance of the mixotroph decreases sharply when carbon input increases, thus allowing a coexistence with heterotrophs at equal densities.

The model reached equilibrium for the full range of values tested, showing that the qualitative results were not unduly sensitive to the parameters; we did not observe complex dynamics (such as limit cycles) in any case. Our results imply that, provided the photosynthetic advantage of the mixotroph is not too large, the heterotrophic mutant will invade a community of mixotrophs and bacteria under a wide range of conditions and may become dominant in both poor and rich environments under certain conditions (Fig. 2): At low carrying capacity, the heterotrophic mutant coexisted with the mixotroph over a wide parameter range, and reached higher densities at low *c_i_* and low *r_M_* values and at high *c_i_* and high *r_M_* (Fig. 2a). The mixotroph strongly dominated only under one condition: at very low *c_i_* and high *r_M_*, which is supported by experimental data [31], but this dominance decreased sharply as *c_i_* levels increased, given that high photosynthetic growth rates imply low levels of heterotrophy. At high *c_i_* and low *r_M_,* in which case the mixotroph retains most of its capacity to capture bacteria, the high carbon supply levels favour large bacteria (the preferred food source of mixotrophs), and both flagellates reached similar densities.

We considered only carbon in our model because bacteria are more often limited by organic carbon than by inorganic nutrients, and small bacteria are comparatively superior to large ones in both respects. Nutrients may play a special role in the development of mixotrophy as a means of additional nutrient uptake for algae in nutrient poor environments, given that bacteria contain high concentrations of P and N [32]. But as the mixotrophic flagellates are no longer nutrient limited, due to their ability to graze bacteria, carbon limited scenarios can be assumed to be more important for explaining the loss of photosynthesis.

## Discussion

Predator-prey size ratio is one of the major factors governing bacteria grazing; rendering ultramicrobacteria too small to be efficiently grazed upon by the (too large) mixotrophic flagellates [19]. However, a drastic reduction of the flagellate's cell size by reduction of the genome and other intracellular structures is hard to achieve as it requires massive reorganization of the cell, including downsizing gene families and protein evolution [34,34]. Similarly, the complete loss of the plastid may not be feasible [35]. Thus, a size reduction of the plastid at the cost of losing some of its functions, specifically photosynthesis, seems a good compromise to decrease cell size and, with that, increase predation efficiency on ultramicrobacteria for mixotrophic algae.

**Figure 3:**
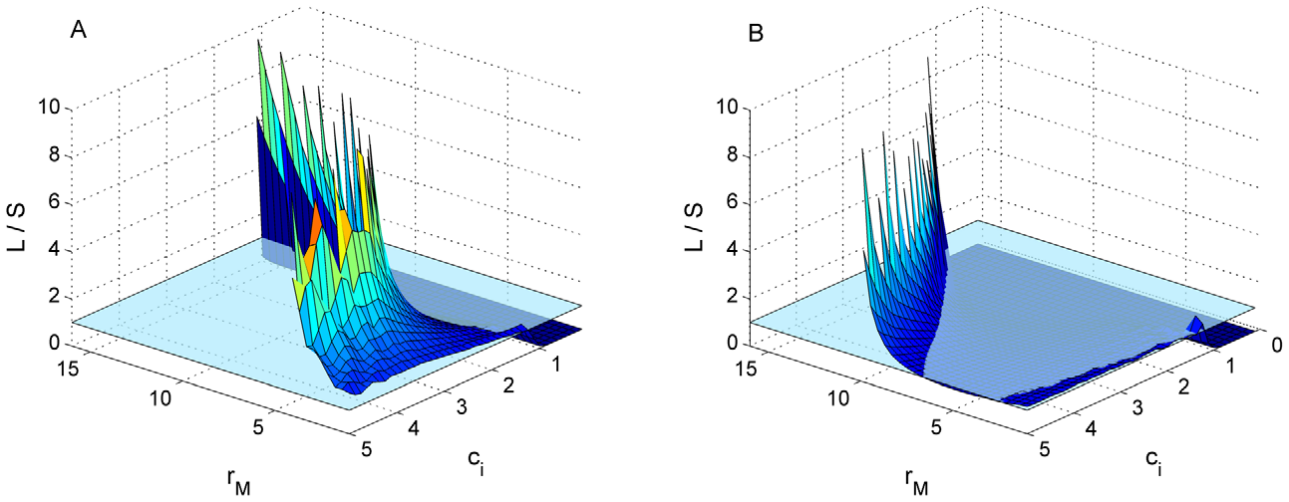
Relative dominance of large vs. small bacteria. Ratio of Large bacteria biomass (L) to Small bacteria biomass (S) at the equilibrium, as a function of carbon input (*c_i_*) and photosynthetic growth rate (*r_M_*×1000). Values above 10 are not plotted. Left panel (A), represents low light availability (*K_M_* = 0.5), while right panel (B) represents high light availability (*K_M_* = 2.5). The horizontal plane marks the 1∶1 ratio. Values higher than 10 are not presented, which causes the “serrated-edge” effect in the figure. The large bacteria dominated over the small ones in richer environments (due to their higher growth rate at higher DOC concentrations), and also as the mixotroph photosynthetic growth rate increased, because the mixotroph grazing pressure (mostly directed to the large bacteria) declines as its photosynthetic ability increases.

Given the results of the model, we can envision two scenarios where the loss of photosynthetic ability (and hence a cell size reduction) could be an advantage and lead to the evolution of heterotrophs. First, in very carbon-poor environments, where small bacteria will strongly dominate over the large ones (Fig. 3) and which are more efficiently exploited by the smaller heterotroph than the larger mixotroph, no matter the photosynthetic advantage of the mixotroph (Fig. 2a). Second, in carbon-rich environments, if the mixotroph has a relatively strong photosynthetic ability because in that case it would have a weak grazing ability, due to the trade-off between grazing and photosynthesis (Fig. 2a). As expected, the advantage of the heterotroph is larger in general if photosynthesis is limited by external factors, for instance low incoming radiation, ice-coverage, or high attenuation. However, even with high light availability, the heterotroph is able to coexist with the mixotroph at similar densities.

As carbon-limited conditions were presumably widespread and typical throughout Earth's history, the first scenario leading to purely heterotrophs within algal lineages under carbon-limited conditions may explain the numerous observed transitions from autotrophic to mixotrophic and further to heterotrophic organisms within virtually all major algal lineages. Our model predicts the evolution of heterotrophs in oligotrophic environments and thereby expands former concepts which associated the evolution of heterotrophy with eutrophic or strongly light-limited environments only.

Our results agree with previous expectations insofar that heterotrophs will dominate in eutrophic environments if photosynthesis is somewhat limited by light availability. However, this scenario is just one possibility out of a variety of scenarios largely determined by the predator-prey interactions between different size classes of bacteria and bacterivorous algae.

Specifically, we do not suggest that an arms race took place between mixotrophic protists and bacteria, each one reducing its size in order to, either escape predation or increase grazing efficiency. In fact, we assume that this kind of arms race already took place between bacteria and pre-existing heterotrophic protists. Under the assumptions of the model, mixotrophs are just faced with the existence of small bacteria as a resource. This is coherent with the fact that photosynthetic flagellates developed much later in evolutionary terms than heterotrophic ones. The newly evolved heterotroph would obviously face competition from previously present heterotrophs. However, the niche of bacterivorous flagellates would not be so fully exploited as to prevent the appearance of new heterotrophic species. More generally, almost any organism that evolves into a new niche will face competition from other species that evolved before, much the same way those species face competition among themselves and also intra-specific competition. The advantage for returning from mixotrophy to heterotrophy is the existence of a resource (small bacteria) that can be grazed more efficiently by reducing size and, ultimately, by losing the photosynthetic apparatus.

Although the structure of our model is very general, the choice of some parameters is designed to reflect the situation in chrysomonad flagellates, since in this group the evolution of phagotrophs within mixotrophic lineages is well documented [5]. However, the results were remarkably robust for a wide range of parameters, and we are confident that the predictions of the model would also hold true for other protists. Thus, we provide a general model explaining the reverse evolution of phagotrophs from mixotrophs in oligotrophic environments without assuming light limitation. Interestingly, size reduction has been discussed as a strategy of bacteria against protistan predators but rarely as a strategy of predators themselves.

## Supporting Information

Figure S1 Ratio of Mixotroph biomass (M) to Heterotroph biomass (H) at the equilibrium, as a function of carbon input (ci) and photosynthetic growth rate (rM×1000), for a3MS increased from 0.15 (as in the main text) to 0.3. This represents a reduction in the advantage of heterotrophs over mixotrophs capturing small bacteria. Left panel (A) is for low light availability (KM = 0.5). Right panel (B) is for high light availability (KM = 2.5). Values higher than 10 are not represented, which causes the gaps along some of the edges in the figure. The horizontal plane marks the 1∶1 ratio.
(1.02 MB TIF)

Text S1 Results of increasing Mixotroph efficiency capturing small bacteria to 0.3.
(0.02 MB DOC)

## References

[1] Cavalier-Smith T (1999). Principles of protein and lipid targeting in secondary symbiogenesis: euglenoid, dinoflagellate, and sporozoan plastid origins and the eukaryote family tree.. J Eukaryot Microbiol.

[2] Sanchez-Puerta MV, Delwiche CF (2008). A hypothesis for plastid evolution in chromalveolates.. J Phycol.

[3] Mallet MA, Lee RW (2006). Identification of three distinct Polytomella lineages based on mitochondrial DNA features.. J Eukaryot Microbiol.

[4] Hachtel W (1998). A plastid genome in the heterotrophic flagellate Astasia longa.. Endocyt Cell Res.

[5] Boenigk J, Pfandl K, Stadler P, Chatzinotas A (2005). High diversity of the “Spumella-like” flagellates: An investigation based on the SSU rRNA gene sequences of isolates from habitats located in six different geographic regions.. Environ Microbiol.

[6] Wilson RJM, Denny PW, Preiser PR, Rangachari K, Roberts K (1996). Complete gene map of the plastid-like DNA of the malaria parasite Plasmodium falciparum.. J Mol Biol.

[7] Nygaard K, Tobiesen A (1993). Bacterivory in algae: a survival strategy during nutrient limitation.. Limnol Oceanog.

[8] Havskum H, Riemann B (1996). Ecological importance of bacterivorous, pigmented flagellates (mixotrophs) in the Bay of Aarhus, Denmark.. Mar Ecol Prog Series.

[9] Thingstad TF, Havskum H, Garde K, Riemann B (1997). On the estrategy of “eating your competitor”: a mathematical analysis of algal mixotrophy.. Ecology.

[10] Raven JA (1997). Phagotrophy in photrotrophs.. Limnol Oceanog.

[11] Troost TA, Kooi BW, Kooijman SALM (2005). Ecological specialization of mixotrophic plankton in a mixed water column.. Am Nat.

[12] González JM (1996). Efficient size-selective bacterivory by phygotrophic nanoflagellates in aquatic ecosystems.. Mar Biol.

[13] Pfandl K, Posch T, Boenigk J (2004). Unexpected effects of prey dimensions and morphologies on the size selective feeding of two bacterivorous flagellates (Ochromonas sp. and Spumella sp.).. J Euk Microbiol.

[14] Hansen B, Bjørnsen PK, Hansen PJ (1994). The size ratio between planktonic predators and their prey.. Limnol Oceanogr.

[15] Boenigk J, Stadler P, Wiedlroither A, Hahn MW (2004). Strain-specific differences in the grazing sensitivity of closely related ultramicrobacteria affiliated with the Polynucleobacter cluster.. Appl Environ Microbiol.

[16] Lanier W, Moustafa A, Bhattacharya D, Comeron JM (2008). EST Analysis of Ostreococcus lucimarinus, the Most Compact Eukaryotic Genome, Shows an Excess of Introns in Highly Expressed Genes.. PLoS ONE.

[17] Sherr EB, Sherr BF (2002). Significance of predation by protists in aquatic microbial food webs.. Anton Leeuw Int J G.

[18] Massana R, Guillou L, Diez R, Pedros-Alio C (2002). Unveiling the organisms behind novel eukaryotic ribosomal DNA sequences from the ocean.. Appl Envrion Microbiol.

[19] Pernthaler J (2005). Predation on procaryotes in the water column and its ecological implications.. Nature Rev Microbiol.

[20] Pernthaler J, Alfreider A, Posch T, Andreatta S Psenner R (1997). In situ classification and image cytometry of pelagic bacteria from a high mountain lake (Gossenköllesee, Austria).. App Environ Microbiol.

[21] Unrein F, Massana R, Alonso-Sáez L, Gasol JM (2007). Significant year-round effect of small mixotrophic flagellates on bacterioplankton in an oligotrophic coastal system.. Limnol Oceanogr.

[22] Boenigk J, Pfandl K, Hansen PJ (2006). Exploring strategies for nanoflagellates living in a ‘wet desert’.. Aquat Microb Ecol.

[23] Hahn MW, Lunsdorf H, Wu QL, Schauer M, Hofle MG (2003). Isolation of novel ultramicrobacteria classified as Actinobacteria from five freshwater habitats in Europe and Asia.. App Environ Microbiol.

[24] Fenchel T, King GM, Blackburn TH (1998). Bacterial biogeochemistry: the ecophysiology of mineral cycling.

[25] Roszak DB, Colwell RR (1987). Survival strategies of bacteria in the natural environment.. Microbiological Reviews.

[26] Giovannoni SJ, Tripp HJ, Givan S, Podar M, Vergin KL (2005). Genome streamlining in a cosmopolitan oceanic bacterium.. Science.

[27] Fegatella F, Lim J, Kjelleberg S, Cavicchioli R (1998). Implications of rRNA operon copy number and ribosome content in the marine oligotrophic ultramicrobacterium Sphingomonas sp. strain RB2256.. App Environ Microbiol.

[28] Klappenbach JA, Dunbar JM, Schmidt TM (2000). RRNA operon copy number reflects ecological strategies of bacteria.. App Environ Microbiol.

[29] Hahn MW (2003). Isolation of strains belonging to the cosmopolitan *Polynucleobacter necessarius* cluster from freshwater habitats located in three climatic zones.. App Environ Microbiol.

[30] Holen DA (1999). Effects of prey abundance and light intensity on the mixotrophic chrysophyte Poterioochromonas malhamensis from a mesotrophic lake.. Freshw Biol.

[31] Katechakis A, Stibor H (2006). The mixotroph Ochromonas tuberculata may invade and suppress specialist phago- and phototroph plankton communities depending on nutrient conditions.. Oecologia.

[32] Stibor H, Sommer U (2003). Mixotrophy of a photosynthetic flagellate viewed from an optimal foraging perspective.. Protist.

[33] Derelle E, Ferraz C, Rombauts S, Rouze P, Worden AZ (2006). Genome analysis of the smallest free-living eukaryote Ostreococcus tauri unveils many unique features.. Proc Natl Acad Sci USA.

[34] Palenik B, Grimwood J, Aerts A, Rouze P, Salamov A (2007). The tiny eukaryote Ostreococcus provides genomic insights into the paradox of plankton speciation.. Proc Natl Acad Sci USA.

[35] Bodył A, Stiller JW, Makiewicz P (2009). Chromalveolate plastids: direct descent or multiple endosymbioses?.. Trends Ecol Evol.

[36] Menden-Deuer S, Lessard EJ (2000). Carbon to volume relationships for dinoflagellates, diatoms, and other protist plankton.. Limnol Oceanogr.

[37] Fenchel T (1986). The ecology of heterotrophic microflagellates.. Adv Microb Ecol.

[38] Fenchel T (1987). Ecology of Protozoa.

[39] Caron DA, Goldman JC, Dennett MR (1990). Carbon utilization by the omnivorous flagellate *Paraphysomonas imperforata*.. Limnol Oceanog.

[40] Boenigk J, Arndt H (2000). Comparative studies on the feeding behavior of two heterotrophic nanoflagellates: the filter-feeding choanoflagellate Monosiga ovata and the raptorial-feeding kinetoplastid Rhynchomonas nasuta.. Aquat Microb Ecol.

[41] Wu QL, Boenigk J, Hahn MW (2004). Successful predation of filamentous bacteria by a nanoflagellate challenges current models of flagellate bacterivory.. App Environ Microbiol.

[42] Pfandl K, Posch T, Boenigk J (2004). Unexpected effects of prey dimensions and morphologies on the size selective feeding of two bacterivorous flagellates (*Ochromonas* sp. and *Spumella* sp.).. J Euk Microbiol.

[43] Gaudy AF, Obayashi A, Gaudy ET (1971). Control of growth rate by initial substrate concentration at values below maximum rate.. Appl Environ Microbiol.

[44] Nedwell DB, Rutter M (1994). Influence of temperature on growth rate and competition between 2 psychrotolerant Antarctic bacteria – Low temperature diminishes affinity for substrate uptake.. Appl Environ Microbiol.

[45] Hahn MW, Lunsdorf H, Wu QL, Schauer M, Hofle MG (2003). Isolation of novel ultramicrobacteria classified as Actinobacteria from five freshwater habitats in Europe and Asia.. App Environ Microbiol.

