# Supplementary figures and images for: Reverse Evolution: Driving Forces Behind the Loss of Acquired Photosynthetic Traits
Source: PLoS One. 2009 Dec 29;4(12):e8465. doi: 10.1371/journal.pone.0008465 (PMC2794545; doi:10.1371/journal.pone.0008465)

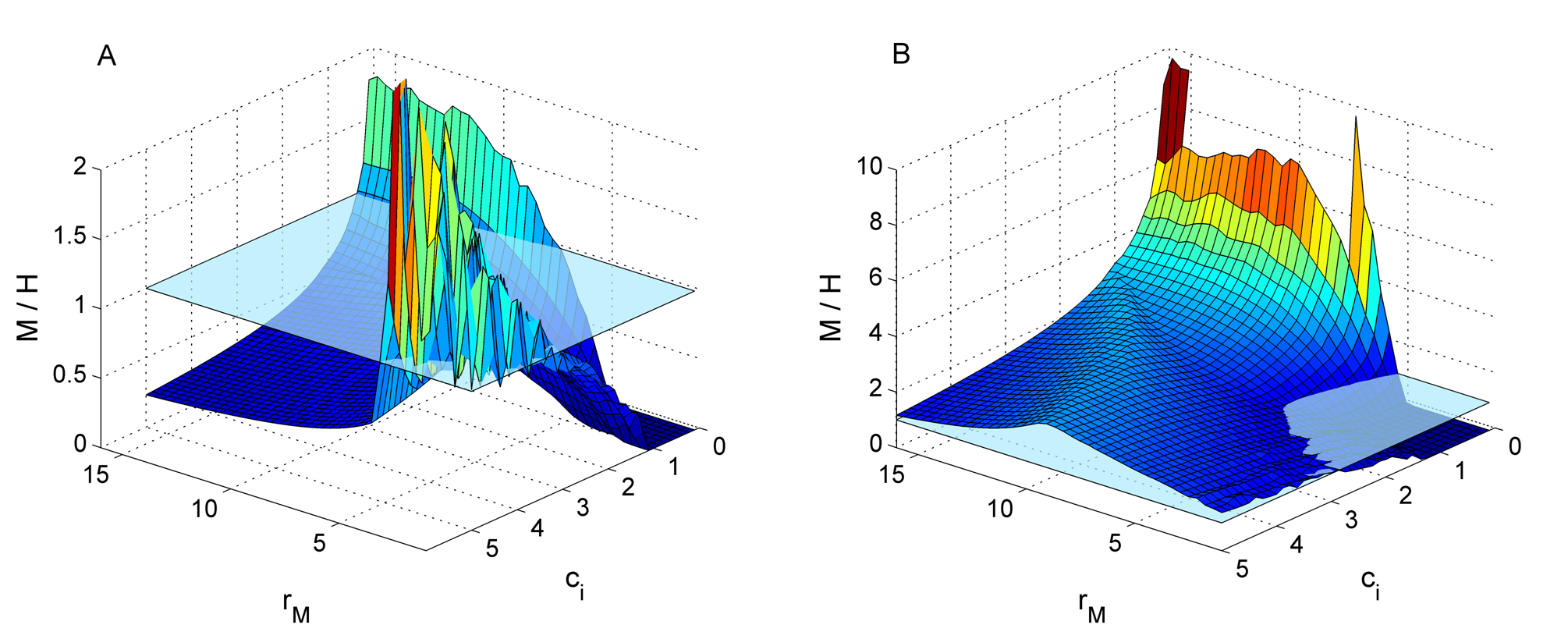

Supplement: Figure S1 — Ratio of Mixotroph biomass (M) to Heterotroph biomass (H) at the equilibrium, as a function of carbon input (ci) and photosynthetic growth rate (rM×1000), for a3MS increased from 0.15 (as in the main text) to 0.3. This represents a reduction in the advantage of heterotrophs over mixotrophs capturing small bacteria. Left panel (A) is for low light availability (KM = 0.5). Right panel (B) is for high light availability (KM = 2.5). Values higher than 10 are not represented, which causes the gaps along some of the edges in the figure. The horizontal plane marks the 1∶1 ratio. (1.02 MB TIF) [file pone.0008465.s001.tif]
