# Supplementary material for: Reverse Evolution: Driving Forces Behind the Loss of Acquired Photosynthetic Traits
Source: PLoS One. 2009 Dec 29;4(12):e8465. doi: 10.1371/journal.pone.0008465 (PMC2794545; doi:10.1371/journal.pone.0008465)
Supplement: Text S1 — Results of increasing Mixotroph efficiency capturing small bacteria to 0.3. (0.02 MB DOC) [file pone.0008465.s002.doc]

**On line Supporting Material**

We tested the consequences of relaxing the assumption that mixotrophs are much less efficient than heterotrophs capturing small bacteria, due to their larger size (5 times larger), which is reflected in the values of a_3MS_ (0.15) and a_3HS_ (0.80) (Table 1). We run additional tests of the model increasing the value of a_3MS_ to 0.3. The results show the same tendency as in the default case, for both low and high light availability. Even if the dominance of the heterotroph decreases somewhat as their advantage grazing small bacteria decreases, the trends are the same, and the heterotroph still dominates in large regions of the parameter space, showing that the model is not so sensitive to this assumption. Moreover, given that grazing efficiency depends on the predator-prey *volume* ratio, rather than mass ratio (see main text for details), a difference of 5 times in mass is ample justification for a ratio of 5 in feeding efficiency used in the main text (0.8/0.15).

**Figure legend**

**Figure S1. Relative dominance of mixotrophs *vs*. heterotrophs.**

Ratio of Mixotroph biomass (M) to Heterotroph biomass (H) at the equilibrium, as a function of carbon input (*c_i_*) and photosynthetic growth rate (*r_M_* x 1000), for α_3MS_ increased from 0.15 (as in the main text) to 0.3. This represents a reduction in the advantage of heterotrophs over mixotrophs capturing small bacteria. Left panel (A) is for low light availability (*K_M_*= 0.5). Right panel (B) is for high light availability (*K_M_*= 2.5). Values higher than 10 are not represented, which causes the gaps along some of the edges in the figure. The horizontal plane marks the 1:1 ratio.
